# The regulatory role of Pcf11-similar-4 (PCFS4) in Arabidopsis development by genome-wide physical interactions with target loci

**DOI:** 10.1186/1471-2164-14-598

**Published:** 2013-09-03

**Authors:** Denghui Xing, Yajun Wang, Ruqiang Xu, Xinfu Ye, Dewei Yang, Qingshun Q Li

**Affiliations:** 1 Department of Botany, Miami University, Oxford, OH 45056, USA; 2 Rice Research Institute, Fujian Academy of Agriculture Sciences, Fuzhou, Fujian, China; 3 Key Laboratory of the Ministry of Education for Coastal and Wetland Ecosystems, and College of the Environment and Ecology, Xiamen University, Xiamen, Fujian 361102, China; 4 Current Address: Department of Biology, Colorado State University, Fort Collins, CO 80526, USA; 5 Current Address: Department of Medicinal Chemistry and Molecular Pharmacology, Purdue University, West Lafayette, IN, USA

**Keywords:** Transcription, Polyadenylation factor, RNA processing, Alternative processing, ChIP-seq

## Abstract

**Background:**

The yeast and human Pcf11 functions in both constitutive and regulated transcription and pre-mRNA processing. The constitutive roles of PCF11 are largely mediated by its direct interaction with RNA Polymerase II C-terminal domain and a polyadenylation factor, Clp1. However, little is known about the mechanism of the regulatory roles of Pcf11. Though similar to Pcf11 in multiple aspects, Arabidopsis Pcf11-similar-4 protein (PCFS4) plays only a regulatory role in Arabidopsis gene expression. Towards understanding how PCFS4 regulates the expression of its direct target genes in a genome level, ChIP-Seq approach was employed in this study to identify PCFS4 enrichment sites (ES) and the ES-linked genes within the Arabidopsis genome.

**Results:**

A total of 892 PCFS4 ES sites linked to 839 genes were identified. Distribution analysis of the ES sites along the gene bodies suggested that PCFS4 is preferentially located on the coding sequences of the genes, consistent with its regulatory role in transcription and pre-mRNA processing. Gene ontology (GO) analysis revealed that the ES-linked genes were specifically enriched in a few GO terms, including those categories of known PCFS4 functions in Arabidopsis development. More interestingly, GO analysis suggested novel roles of PCFS4. An example is its role in circadian rhythm, which was experimentally verified herein. ES site sequences analysis identified some over-represented sequence motifs shared by subsets of ES sites. The motifs may explain the specificity of PCFS4 on its target genes and the PCFS4*'*s functions in multiple aspects of Arabidopsis development and behavior.

**Conclusions:**

Arabidopsis PCFS4 has been shown to specifically target on, and physically interact with, the subsets of genes. Its targeting specificity is likely mediated by cis-elements shared by the genes of each subset. The potential regulation on both transcription and mRNA processing levels of each subset of the genes may explain the functions of PCFS4 in multiple aspects of Arabidopsis development and behavior.

## Background

Gene transcription and pre-mRNA processing are two major processes in eukaryotic mRNA biosynthesis. RNA processing events were thought to follow transcription. However, studies in the past two decades have well established that the pre-mRNA processing is highly co-transcriptional *in vivo*[1]. Pcf11 (Protein 1/Cleavage Factor 1) is one of such proteins that couple pre-mRNA processing with transcription [2-5].

Originally identified as a factor required for pre-mRNA 3′-end processing and transcription termination [2,6], Pcf11 was also eventually found to play a role in transcription initiation, elongation and mRNA export from nucleus to cytoplasm [7-10]. Recent studies revealed additional roles of Pcf11 in transcription termination of snRNA, snoRNA and cryptic unstable transcripts [8,11]. The effects of Pcf11 on transcription and pre-mRNA processing are largely mediated by its interactions with RNA polymerase II C-terminal domain (Poly II CTD) and other polyadenylation factors [3-5,12-15]. Disruption of these interactions led to altered transcription termination and decreased polyadenylation efficiency [12,13]. The interactions could be affected directly by the phosphorylation status of Pol II CTD and indirectly by cis-elements within pre-mRNA, possibly through RNA-binding factors (e.g. RNA15 and Hrp1) [6,16-21].

Given the key role of Pcf11 in coupling transcription termination and 3′end processing, it is not surprising that Pcf11 also serves as a target for regulated transcription and pre-mRNA processing. Recent studies revealed its additional roles, direct and indirect, in regulated transcription initiation, elongation, termination and alternative processing of pre-mRNA [7,8,10,15,22].

PCFS4 is one of the Arabidopsis orthologs of yeast and human Pcf11 [23]. While similar to Pcf11 in both its amino acid sequence and domain structures, PCFS4, unlike its yeast or human counterpart, was not required for the viability of Arabidopsis plants [23]. Functional characterizations revealed the role of PCFS4 in Arabidopsis development such as flowering time [23]. Molecular characterizations suggested that the function of PCFS4 in flowering control was partially mediated by the alternative processing (AP) of *FCA*, a gene encoding a flowering time regulator [23]. However, the AP of *FCA* could not fully account for the delayed flowering of *pcfs4* mutants, nor was it responsible for the other developmental defects, suggesting that there must be gene(s) other than *FCA* being targeted by PCFS4 [23]. Supporting this hypothesis were the hundreds of differentially expressed genes in the *pcfs4-1* mutant revealed by genome-wide gene expression profiling [24].

Given that Pcf11 was recruited to actively transcribed gene loci through interaction with Pol II CTD [3,25], we hypothesize that PCFS4 may also physically interact with the loci of its direct targets. To test this hypothesis, we employed a ChIP-Seq assay to identify PCFS4 enriched sites (ES) within the Arabidopsis genome. The results shed light on what genes were directly targeted and how PCFS4 might be recruited to the loci of those targets. Gene ontology (GO) analysis of the ES-linked genes revealed enriched GO terms that both explained the known developmental defects of *pcfs4* mutants and suggested additional regulatory roles of PCFS4 in other biological processes.

## Results

### PCFS4 functions in multiple aspects of Arabidopsis development

**Figure 1:**
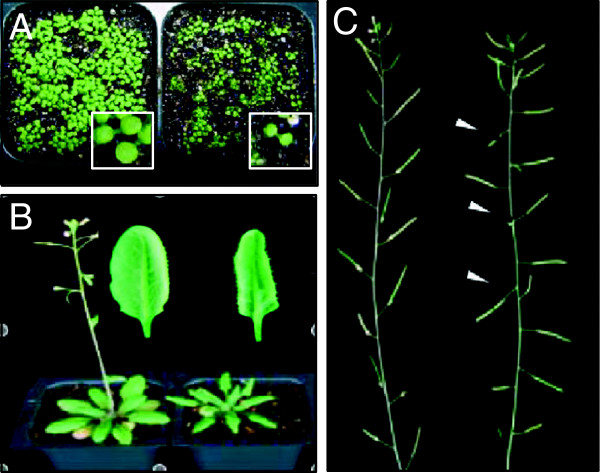
**The pleiotropic effects of *pcfs4 *mutant on Arabidopsis development.** Shown on individual panels are wild type Col (left hand side) and mutant *pcfs4-1* (right hand side). The mutant showed a variety of aberrant morphologies including smaller seedlings **(A)**, down-curved leaf shape, delayed flowering **(B)**, and altered inflorescence phyllotaxy **(C)**. The embedded seedlings within panel A were a detailed look with equal magnification. The arrows within panel C point to the siliques forming a larger angle with the stem. Pictures were taken at 10, 40, 50 days after germination, respectively, for **(A)**, **(B)** and **(C)**.

Previously, we have genetically and molecularly characterized the functions of PCFS4 in flowering time control. We found that the flowering delay of the *pcfs4* mutants could be partially explained by the effects of PCFS4 on the alternative processing of *FCA* pre-mRNA [23]. In addition to its delayed flowering, *pcfs4* mutants also showed other phenotypes including reduced vigor of their seedlings, altered leaf shape and inflorescence phyllotaxy (Figure 1). In the early seedling stage, mutant plants were significantly smaller than the wild type (Figure 1A). Leaf edges of *pcfs4-1* were more curved down towards its abaxial side (Figure 1B). The siliques of *pcfs4-1* mutants formed a larger angle with its stem in contrast to that in wild type Col (Figure 1C). Albeit all these defects, the mutant remained quite healthy throughout its whole life cycle in the standard growth conditions, suggesting that PCFS4 is not essential for the viability of the plants. Supporting this conclusion is that the expression of PCFS4 is not ubiquitous, but instead, tissue-specific and developmentally regulated [23]. The non-essential nature of PCFS4 for plant viability and specific developmental defects of *pcfs4* mutants support a hypothesis that PCFS4 might specifically target on a subset of genes.

### PCFS4 interacts with Arabidopsis Pol II CTD domain

**Figure 2:**
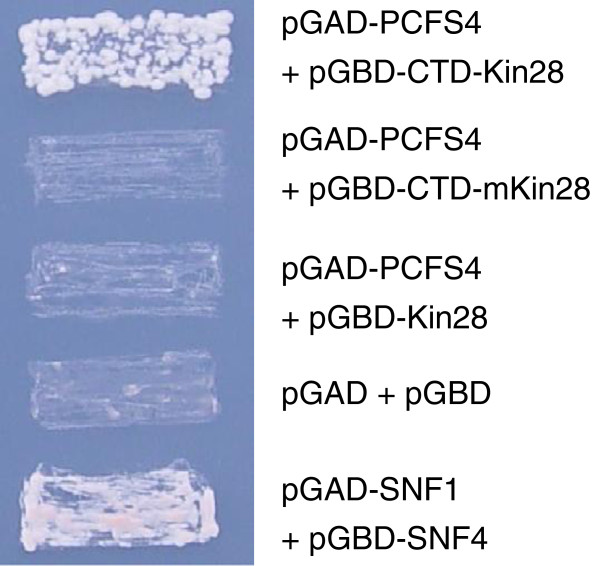
**PCFS4 interacts with phosphorylated Pol II CTD domain.** Yeast two-hybrid analyses of pair-wise interactions of PCFS4/CTD-Kin28, PCFS4/CTD-mKin28 and PCFS4/Kin28. AD, activation domain; BD, binding domain; CTD, RNA polymerase II C-terminal domain; mKin28 indicates a mutated Kin28 kinase dormain. The vector plasmids pGAD/pGBD were used as a negative control while pAD-SNF1/BD-SNF4 was a positive control.

In mammals and yeast, Pcf11 is recruited to actively transcribed gene loci through its interaction with phosphorylated Pol II CTD domain [3,25]. The interaction plays a pivotal role in mediating the effects of Pcf11 on both transcription and pre-mRNA processing [3-5,12,13]. To examine whether PCFS4 interacts with Arabidopsis Pol II CTD, we carried out a modified yeast two-hybrid assay (Y2H) [26]. In the assay, the bait contained the Kin28-tethered Arabidopsis Pol II CTD domain. Kin28 is a protein kinase and tethering it with the CTD ensures that the CTD is phosphorylated [26,27]. The results of Y2H assays indicated that PCFS4 did interact with the CTD-Kin28. And since no interaction was detectable when Kin28 was mutated (Figure 2), we concluded that the interaction was dependent on the phosphorylation of the CTD. To rule out that the interaction might be attributed to kin28 portion of the fusion protein, the interaction between PCFS4 and Kin28 alone was tested, with the result being that no interaction was detectable (Figure 2). Taken together, these results indicated that PCFS4 did interact with phosphorylated Pol II CTD, further supporting the idea that PCFS4 might be recruited to the loci of its direct targets.

### PCFS4-TAP fusion protein was enriched on hundreds of genomic regions

To address if PCFS4 physically interact with its target genes and what these genes might be, we transformed the *pcfs4-1* mutant with a transgene encoding PCFS4-TAP (Tandem Affinity Purification) fusion protein. The transgene successfully complemented the mutant phenotypes and the expression of the fusion protein was confirmed by western blots using the peroxidase-conjugated anti-peroxidase antibody against the TAP tag (Additional file 1: Figure S1). We then performed a ChIP (chromatin immunoprecipitation) using the same antibody following the formaldehyde cross-linking treatment of two-week old seedlings. The ChIP DNA and the input DNA were further sequenced using Illumina sequencing platform.

The sequence reads (75-base long) derived from Illumina sequencing were first mapped to the Arabidopsis genome using Bowtie [28]. About 2 million and 4.2 million of reads from ChIP and input samples, respectively, were successfully mapped to the genome. The mapped reads were further analyzed using Cisgenome to identify the enrichment sites (ES) that were over-represented in the sequence reads from the ChIP sample [29]. The input sample was used as a background control in this analysis. 892 ES sites were identified with the following criteria: Log2 Fold Change ≥ 5; p-value ≤ 0.001; and the false discovery rate (FDR) = 0.02 (Additional file 2: Table S1).

**Figure 3:**
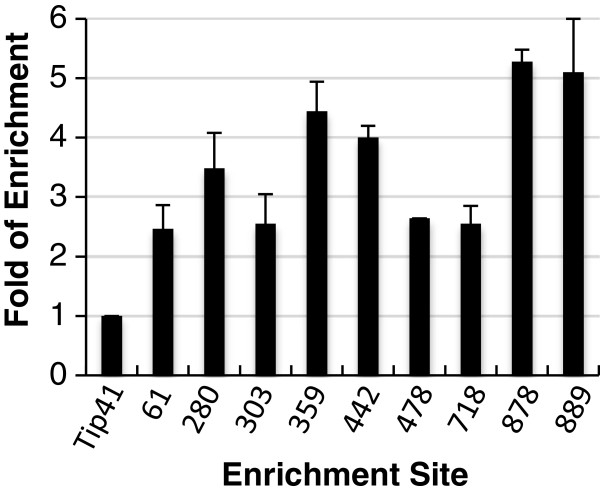
**Verification of the PCFS4-TAP enrichment on the enriched site regions by qPCR.** Following the ChIP using an antibody against the PCFS4-TAP fusion protein, the DNA abundance (mean ± stdev) of the PCFS4-TAP enriched sites was determined by using qPCR and normalized to the PCFS4-TAP enrichment on the loci of Tip41 [30]. The numbers on the X-axis indicate the rankings of the enriched site as detailed in Additional file 2: Table S1.

To verify the enrichment of PCFS4-TAP on the identified ES, we performed a real-time PCR (qPCR) analysis following the ChIP for 9 randomly selected ES sites, which cover the entire ES list with p-value ranking from low to high (Additional file 2: Table S1). The results indicated that PCFS4-TAP was indeed enriched on all tested ES sites in the ChIP sample. The enrichment ranged from 2 to 5 fold, relative to the control sites (Figure 3). Thus, the identified ES were truly representing the PCFS4-TAP enrichment sites within Arabidopsis genome.

**Figure 4:**
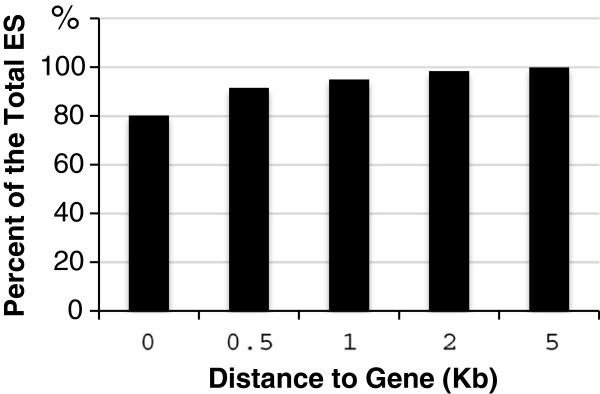
**The distribution of enriched sites (ES) within and around the genes.** Shown is the percent of the ES falling between transcription start site (TSS) and transcription ending site (TES) (0); between TSS-500 bp and TES + 500 bp (0.5); between TSS-1 kb and TES + 1 Kb (1); between TSS-2 kb and TES + 2 Kb (2); between TSS-5 kb and TES + 5 Kb (5); between TSS-1 kb and TES + 1 Kb (1).

When the distribution of the ES was examined, it was found that 80% of the ES were located on intragenic regions (Table 1). For the ES on intergenic regions, the majority of them were located in the vicinity of genes. 95% of ES were located either within genes or 1 kb up- or down-stream of the genes (Figure 4). Thus, PCFS4 was preferentially located on the intragenic region, consistent with its potential role in regulating transcription and pre-mRNA processing.

**Table 1:** The distribution of the enriched sites (ES)

**Location**			**No. ES**	**Subtotal ES (%)**	**Total ES (%)**
Intergenic			177		19.8
Intragenic	Exon	5*'*UTR	33	5	65.7
		CDS	485	81	
		3*'*UTR	82	14	
	Intron		129		14.5
Total			892		100

When the ES distribution within the intragenic region was considered, 66% of the ES were located within exons, while 15% of them were located within introns (Table 1). Of the ES within exons, there are 5% within 5′UTR; 81% within coding sequence region (CDS); and 14% within 3′UTR (Table 1). Thus, PCFS4-TAP was predominantly enriched within CDS region.

### Identification of common cis-elements

**Figure 5:**
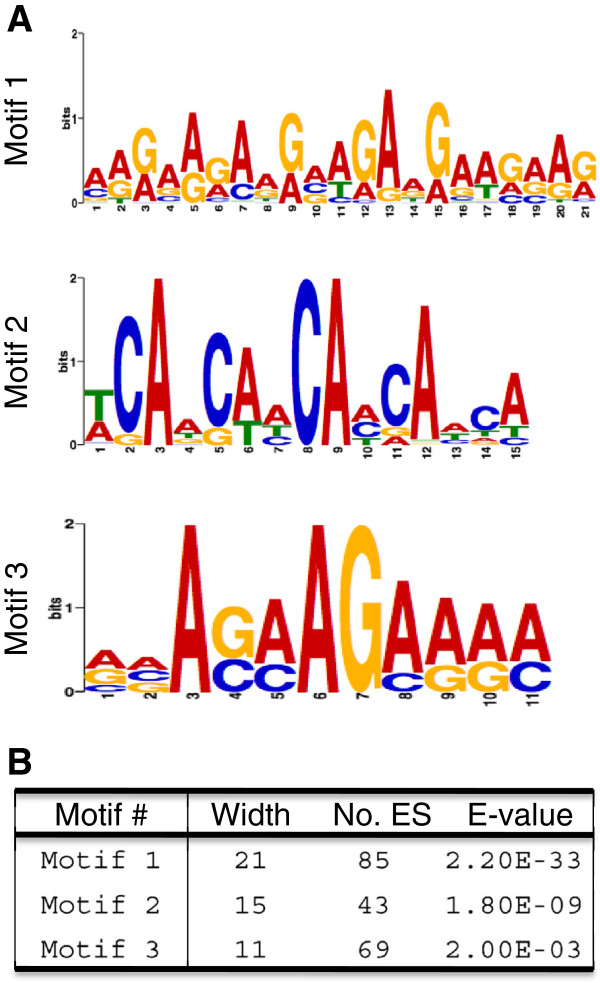
**The sequence motifs identified from ES sequences.** Shown are the sequence logos of Motif 1, Motif 2 and Motif 3 **(A)** and their Width (Nucleotide No.), the number of covered ES sites (No. ES) and their significance level (E-value) **(B)**.

With the identified ES, we reasoned that there could exist a unique, shared cis-element(s) rendering these ES to be specifically targeted by PCFS4, either directly or indirectly [through other factor(s)]. To explore the potential cis-element(s), we analyzed the sequences of these ES using MEME-ChIP program [31]. Three sequence motifs with varied significance, ranging from 2.2x10^-33^ to 2.0x10^-3^, were identified (Figure 5). Motif 1 was 21 bp long and shared by 85 ES sites; motif 2 was 15 bp long and shared by 43 ES sites; and motif 3 was 11 bp long and shared by 69 ES sites (Figure 5). However, not all ES sites (892) were covered by the 3 motifs, suggesting additional motifs that could not be identified by this method.

### GO enrichment analysis of ES-linked genes

To explore the biological significance of PCFS4 enrichments on the identified ES, the ES-linked genes were extracted and analyzed. An ES-linked gene is defined as one that is closest to a given ES or an ES within 2 Kb up-stream of the gene’s start codon and 2 Kb down-stream of its stop codon. 839 such genes were identified, with a majority of them being linked with a single ES (821 or 98%) and 18 of them (2%) linked with 2 ES (Additional file 2: Table S1).

With the ES-linked genes as inputs, GO enrichment analyses were performed using GeneCodis [32]. The results revealed that multiple GO terms were over-represented in the ES-linked genes (p-value ≤ 0.01; FDR ≤ 0.05) (Table 2; Additional file 2: Table S2; Table S3). A similar set of enriched GO terms was identified when the genes were analyzed using GOEAST, another GO enrichment analysis tool (Additional file 2: Table S4) [33]. Among those GO terms were “photomorphogenesis”, “Embryo development ending in seed dormancy” and “negative regulation of flower development”, which are consistent with the known altered morphologies and delayed flowering of *pcfs4-1* mutants (Figure 1; Table 2; Additional file 2: Table S3). The other enriched GO terms include circadian rhythm, gametophyte development, protein kinase activity, plant cell wall synthesis, and response to fungus, suggesting potential roles of PCFS4 in additional biological processes (Table 2). These results implied that PCFS4 might serve as an important regulator of transcription and pre-mRNA processing for genes involved in a variety of biological functions in plants.

**Table 2:** Enriched GO terms identified with GeneCodis

**GO ID**	**Ontology#**	**Term**	**Num. of**	**p-value**	FDR*
			**Genes**		
GO:0016049	BP	cell growth	7	3.4E-06	3.5E-04
GO:0005086	MF	ARF guanyl-nucleotide exchange factor activity	4	5.5E-06	6.4E-04
GO:0005488	MF	binding	36	6.3E-05	2.4E-03
GO:0009640	BP	photomorphogenesis	7	1.5E-04	5.4E-03
GO:0009555	BP	pollen development	11	2.5E-04	6.4E-03
GO:0016192	BP	vesicle-mediated transport	10	6.7E-04	1.4E-02
GO:0042752	BP	regulation of circadian rhythm	4	7.1E-04	1.2E-02
GO:0004553	MF	hydrolyzing O-glycosyl compounds	16	7.7E-04	1.5E-02
GO:0009793	BP	embryo development ending in seed dormancy	19	1.0E-03	1.3E-02
GO:0004672	MF	protein kinase activity	28	1.1E-03	1.9E-02
GO:0080167	BP	response to karrikin	10	1.3E-03	1.4E-02
GO:0050790	BP	regulation of catalytic activity	4	1.4E-03	1.2E-02
GO:0009561	BP	megagametogenesis	4	1.4E-03	1.2E-02
GO:0003700	MF	sequence-specific DNA binding transcription factor activity	61	2.1E-03	3.1E-02
GO:0005524	MF	ATP binding	52	2.1E-03	2.8E-02
GO:0003677	MF	DNA binding	53	2.9E-03	3.4E-02
GO:0008270	MF	zinc ion binding	48	3.7E-03	3.6E-02
GO:0050832	BP	defense response to fungus	9	5.4E-03	4.3E-02
GO:0009832	BP	plant-type cell wall biogenesis	4	5.6E-03	4.1E-02
GO:0015031	BP	protein transport	8	6.4E-03	4.4E-02
GO:0004871	MF	signal transducer activity	8	6.4E-03	4.9E-02
GO:0044237	BP	cellular metabolic process	4	7.9E-03	4.9E-02
GO:0000226	BP	microtubule cytoskeleton organization	3	8.1E-03	4.7E-02

Note: #: *BP*, Biological Process; *MF*, Molecular Function.

*: *FDR*, False Discovery Rate.

### PCFS4 plays a role in Arabidopsis circadian rhythm

To find further supporting evidence for the implied roles of PCFS4 in “additional” biological processes, we focused on the genes within the enriched GO term “regulation of circadian rhythm”. The GO term contains 5 ES-linked genes (Additional file 2: Table S5). The enrichment of PCFS4 on these loci was verified by qPCR (Additional file 1: Figure S2). Since PCFS4 was an ortholog of Pcf11 and known to play a role in alternative pre-mRNA processing [23], we examined the alternative processing profiles of these genes using publically available cDNA/EST data (TAIR10). Indeed, all five genes showed certain forms of alternative transcription and/or pre-mRNA processing evidenced by the cDNA and/or EST data (Additional file 1: Figure S3). Most importantly, the ES sites on these genes were often associated with the positions where the alternative processing or alternative transcription initiation occurred (Additional file 1: Figure S3).

**Figure 6:**
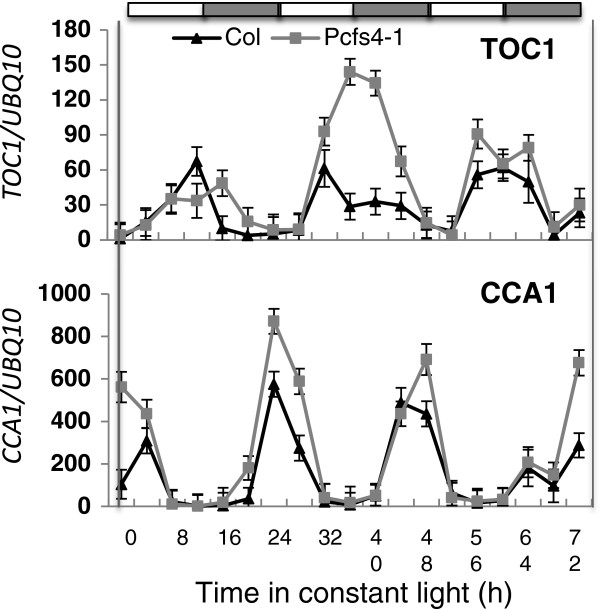
**The *pcfs4 *mutation affects the period and amplitude of expression of *TOC1 *and *CCA1. ***Col and *pcfs4-1* seedlings were entrained to photocycles (Light/Dark: 12/12 h) for 10 days before release into constant light. Transcript abundance (mean ± stdev) was estimated by qPCR and normalized to ubiquitin (UBQ10) expression. White and gray bars indicate subjective day and night, respectively.

We further tested the effects of *PCFS4* mutation on circadian rhythm in Arabidopsis by quantifying the expression of *TOC1* and *CCA1*, two key circadian clock genes [34,35]. As shown in Figure 6, both the amplitude and circadian period were altered for the expression of *TOC1* and *CCA1*, with the *pcfs4* mutant showing a lengthened circadian period and increased amplitude for both genes. Thus, PCFS4 indeed played a role in Arabidopsis circadian rhythm, likely through direct and indirect regulation of the transcription and/or pre-mRNA processing of its target genes.

## Discussion

Accumulating evidence in all three kingdoms of eukaryotic organisms supports the idea that alternative processing of pre-mRNA plays a key role in regulation of gene expression and the transcriptome complexity [36-41]. Among the many factors affecting AP of pre-mRNA, one is Pcf11. While essential for pre-mRNA 3′ end processing and transcription termination in general, there was evidence that Pcf11 played a regulatory role in the pre-mRNA processing of some genes [15,22,42,43]. Being one of Arabidopsis orthologs of Pcf11, PCFS4 was of special interest in that it is, unlike Pcf11, not essential for the plant viability [23]. The non-essential nature and the pleiotropic effects of PCFS4, together with its proved regulatory role in *FCA* pre-mRNA processing, argue that PCFS4 may have specifically adapted itself for regulated pre-mRNA processing of a subset of genes in plants. However, how PCFS4, like Pcf11 when playing a regulatory role, gains its specificity remains elusive.

The interaction of PCFS4 with Pol II CTD provides evidence that PCFS4 was recruited to actively transcribed gene loci (Figure 2). This interaction, on the other hand, does not define the target genes by PCFS4 since Pol II CTD is universally required for all actively transcribed protein-coding genes. Towards understanding how PCFS4 might gain its target specificity, we identified the genome-wide PCFS4-TAP ES sites that were specifically concentrated on the genic regions (Table 1). Bioinformatic analysis identified a few unique sequence motifs that were shared by some of the ES sites (Figure 5). These sequence motifs could be essential elements providing PCFS4 target specificity either as cis-elements within genes or pre-mRNAs. So, how are the cis-element-containing genes specifically targeted by PCFS4? One scenario could be that the cis-elements within the pre-mRNA compete with Pol II CTD for binding PCFS4 so that the interaction between PCFS4 and CTD is disrupted, leading to an altered pre-mRNA processing. Evidence supporting this scenario is the weak RNA binding activity of Pcf11 and the competitive Pcf11-binding between RNA and Pol II CTD [17]. Alternatively, the cis-elements within the gene may affect the phosphorylation status of Pol II CTD, leading to gene-specific CTD code(s), which again may influence the CTD-PCFS4 interaction [18].

The predominant location of ES on the CDS region was surprising, given that Pcf11 in yeast was preferentially mapped to the 3′ end of the gene loci [18-21]. However, this discrepancy might well explain the non-essential nature of PCFS4. In other words, PCFS4 may mainly play a regulatory role for transcription and pre-mRNA processing of a subset of genes while its yeast ortholog, pcf11, acts mainly as a general transcription termination and 3′end pre-mRNA processing factor [20,21].

The GO enrichment analysis of ES-linked genes revealed the functions of PCFS4 beyond what we have known previously. Not only were the enriched GO terms consistent with PCFS4′s functions in Arabidopsis development and flowering control but also revealed its potential roles in circadian rhythm, response to fungus pathogen and plant cell wall synthesis (Table 2). We were also able to verify PCFS4′s effects on Arabidopsis circadian rhythm and the coincidence of PCFS4 ES sites with the sites where the alternative processing was suggested by cDNAs and/or ESTs. This shined light on how PCFS4 functions in this biological process. Interestingly, recent studies also revealed a significant role of pre-mRNA alternative processing in regulating the expression of circadian clock genes [44-47]. Our discovery offers additional evidence of such a regulation.

These results, together with what have been known about PCFS4 and Pcf11, lead to a conceivable model by which the biological functions of PCFS4 might be explained. In this model, PCFS4 is recruited to the loci of subsets of genes. Each subset of genes, whose regulated expression mediates a specific biological effect of PCFS4, shares a common cis-element. The cis-element, when existing in pre-mRNA, may affect the PCFS4-CTD interaction by competitively binding PCFS4 with CTD, or by recruiting another PCFS4-binding factor [14,17]. Alternatively, when present on the gene, the cis-element may recruit factors affecting the phophorylation status of Pol II CTD domain [18]. By either way, the PCFS4-CTD interaction will be affected, leading to altered gene transcription and/or pre-mRNA processing. Depending on the functional nature of each subset of genes, the cis-element and its relative locations on the genes (5′ end, 3′ end or middle section of the gene) could vary. The protein factors mediating the cis-element’s function may be unique for each subset of genes. The combination of the cis-elements, their locations and the mediating factors may explain the multiple biological effects of PCFS4.

## Conclusions

It is demonstrated that Arabidopsis PCFS4 specifically targets subsets of genes. Its targeting specificity is likely mediated by the cis-element shared by the genes of each subset. The potential regulation at the level of transcription and mRNA processing may be the basis for its multi functions in different aspects of Arabidopsis development and environmental responses. The targeting specificity of Arabidopsis PCFS4 might also suggest a potential mechanism of human and yeast Pcf11 in regulating gene transcription and mRNA processing.

## Methods

All *Arabidopsis thaliana* plants used in this study are in Col background. The yeast strains, the pGAD-PCFS4 construct and control plasmids for Y2H assay had been described previously [23]. The pGBD-CTD-Kin28, pGBD-CTD-mKin28, and pGBD-Kin28 were kind gifts from Dr. Hisashi Koiwa (Texas A&M University). The Y2H assay was performed as described previously [23].

Arabidopsis seeds were germinated and grown on SunGrow 360 soil under standard conditions as described previously [23]. Plant pictures were taken at different growth stages suitable for each phenotype as indicated.

The ChIP assay was carried out largely based on the published protocols with slight modification [48,49]. Briefly, Transgenic seeds containing *PCFS4-TAP* transgene were germinated on MS medium at 4°C in dark for 2 days and then moved to a grow chamber with 22°C, 16/8 hr light/dark cycles. Two-week old seedlings were harvested and cross-linked with 1% formaldehyde and further processed as described [48]. The chromatin was sheared by sonication to 300–1000 bp fragments. The sample was centrifuged and the supernatant was transferred to two siliconized tubes, one for immuno-precipitation (IP) and the other as an input control (IN). For the IP sample, 60 μl sepharose IgG beads was added and incubated for 3 hr at 4°C. The sample was washed and the bead-binding complexes were eluted with elution buffer [48]. The IN sample and the eluted IP sample were treated with 200 mM NaCl to reverse the cross-linking. The samples were digested by proteinase K to remove proteins, treated with phenol/chloroform extraction, and DNA fragments were recovered by ethanol precipitation. The enrichment of DNA on tested genomic regions was estimated using real-time PCR (qPCR). The oligonucleotide primers used for detecting each ES site or ES sites within genes were listed in Additional file 2: Table S6.

For the ChIP-Seq assay, the ChIP samples were prepared essentially the same as described above except that the chromatin was sheared to 100 to 500 bp long fragments. The precipitated DNA was further processed following the instruction of Illumina ChIP-Seq DNA Sample Prep Kit (Illumina Inc). The DNA library was sequenced using Illumina platform Genome Analyzer II in the Ohio State University MCIC (Wooster, Ohio). The sequencing reads were mapped to the Arabidopsis genome (TAIR10; http://www.arabidopsis.org) using Bowtie with the following mapping parameters: the quality matrix, phred64-quals; the minimum seed length, l = 20; the allowed mismatch in the seed, n = 1 [28]. The mapped reads (both IP and IN) were used as input to analyze the significantly enriched peaks using Cisgenome with default parameters [29]. The sequences of the enriched peak sites and their linked genes were extracted using the same program package. The sequence data (.fastq files) were deposited to The NCBI Sequence Read Archive with accession number of SRA060798.

For sequence motif identifications, the enriched peak sites were analyzed using MEME-CHIP to find over-represented sequence motifs with the parameter setting: Distribution of motif occurrences, zero or one per sequence; Minimum motif width, 6; Maximum motif width, 30 [31]. For the GO enrichment analysis, the ES-linked genes were analyzed using GeneCodis and GOEAST program packages [32,33].

For circadian rhythm analysis, the seeds of Col and *pcfs4-1* mutant were germinated on MS medium at 4°C in the dark for 2 days and moved to growth chamber under 22°C, 12/12 hr light/dark photoperiod. Ten days later, the chamber was set on constant light conditions. The seedlings were collected every four hours starting right at the beginning of the constant light and ending at 72 hrs. The collected seedlings were immediately frozen in liquid nitrogen and stored at -80°C until all the samples were collected. Total RNA was extracted using Concert™ Plant RNA Reagent and treated with Turbo DNase-free (both from Invitrogen). The DNase treated RNA was reverse-transcribed using SuperScript^®^ III (Invitrogen) and Oligo-dT(18) primer. The abundance of *CCA1* and *TOC1* transcripts were estimated with qPCR and normalized to the abundance of *UBQ10* transcripts. The primers used for the qPCR are listed in Additional file 2: Table S6.

## Competing interests

The authors declare that they have no competing interests.

## Authors’ contributions

QQL and DX conceived the experiments; DX, RX, XY, DY carried out the experiments; DX, YW analyzed the data; DX and QQL wrote the manuscript. All authors read and approved the final manuscript.

## Supplementary Material

Additional file 1: Figure S1 Expression of PCFS4-TAP fusion protein in transgenic plants. The total protein extracts from the mutant (*pcfs4-1*) and the mutant containing the gene construct of *35S: PCFS4-TAP* (*PCFS4-TAP*) were fractioned, blotted to membrane and immuno-detected using peroxidase-conjugated anti-peroxidase IgG against the TAP tag (upper-panel). The Coomassie blue stained gel image (lower-panel) showed an equal loading for the two samples. **Figure S2.** Verification of the PCFS4-TAP enrichment on the gene loci involved in circadian rhythm. Following the ChIP, the DNA abundance (mean ± stdev) of the PCFS4-TAP enriched sites were determined using qPCR and normalized to the PCFS4-TAP enrichment on Tip41 (the control). **Figure S3.** PCFS4-TAP enrichment sites are associated with the sites where the alternative transcription or pre-mRNA processing occurs. The gene structures are represented by blue bars (light blue bars for 5*'* and 3*'* UTR) and lines (Gene model). The cDNA/ESTs supporting the gene model are represented by green bars (cDNA/EST). The vertical black bars represent the PCFS4-TAP enrichment (Log fold enrichment) along the gene body. The red frames highlight the regions where the ES site are associated with alternative transcription or pre-mRNA processing supported by cDNA/ESTs. Click here for file

Additional file 2: Table S1 The PCFS4‒TAP enrichment site (ES) and their linked genes. **Table S2.** Enriched GO terms identified with GeneCodis and the genes within the GO terms. **Table S3.** Group of coherent GO terms generated through GeneTerm Linker. **Table S4.** Enriched GO terms identified using GOEAST. **Table S5.** ES-linked Genes within the enriched GO term “Regulation of Circadian Rhythm” (GO:0042752). **Table S6.** The primers used in this study. Click here for file
