# Supplementary material for: The regulatory role of Pcf11-similar-4 (PCFS4) in Arabidopsis development by genome-wide physical interactions with target loci
Source: BMC Genomics. 2013 Sep 3;14:598. doi: 10.1186/1471-2164-14-598 (PMC3844406; doi:10.1186/1471-2164-14-598)
Supplement: Additional file 1: Figure S1 — Expression of PCFS4-TAP fusion protein in transgenic plants. The total protein extracts from the mutant (pcfs4-1) and the mutant containing the gene construct of 35S: PCFS4-TAP (PCFS4-TAP) were fractioned, blotted to membrane and immuno-detected using peroxidase-conjugated anti-peroxidase IgG against the TAP tag (upper-panel). The Coomassie blue stained gel image (lower-panel) showed an equal loading for the two samples. Figure S2. Verification of the PCFS4-TAP enrichment on the gene loci involved in circadian rhythm. Following the ChIP, the DNA abundance (mean ± stdev) of the PCFS4-TAP enriched sites were determined using qPCR and normalized to the PCFS4-TAP enrichment on Tip41 (the control). Figure S3. PCFS4-TAP enrichment sites are associated with the sites where the alternative transcription or pre-mRNA processing occurs. The gene structures are represented by blue bars (light blue bars for 5' and 3' UTR) and lines (Gene model). The cDNA/ESTs supporting the gene model are represented by green bars (cDNA/EST). The vertical black bars represent the PCFS4-TAP enrichment (Log fold enrichment) along the gene body. The red frames highlight the regions where the ES site are associated with alternative transcription or pre-mRNA processing supported by cDNA/ESTs. [file 1471-2164-14-598-S1.pptx]

## Slide 1
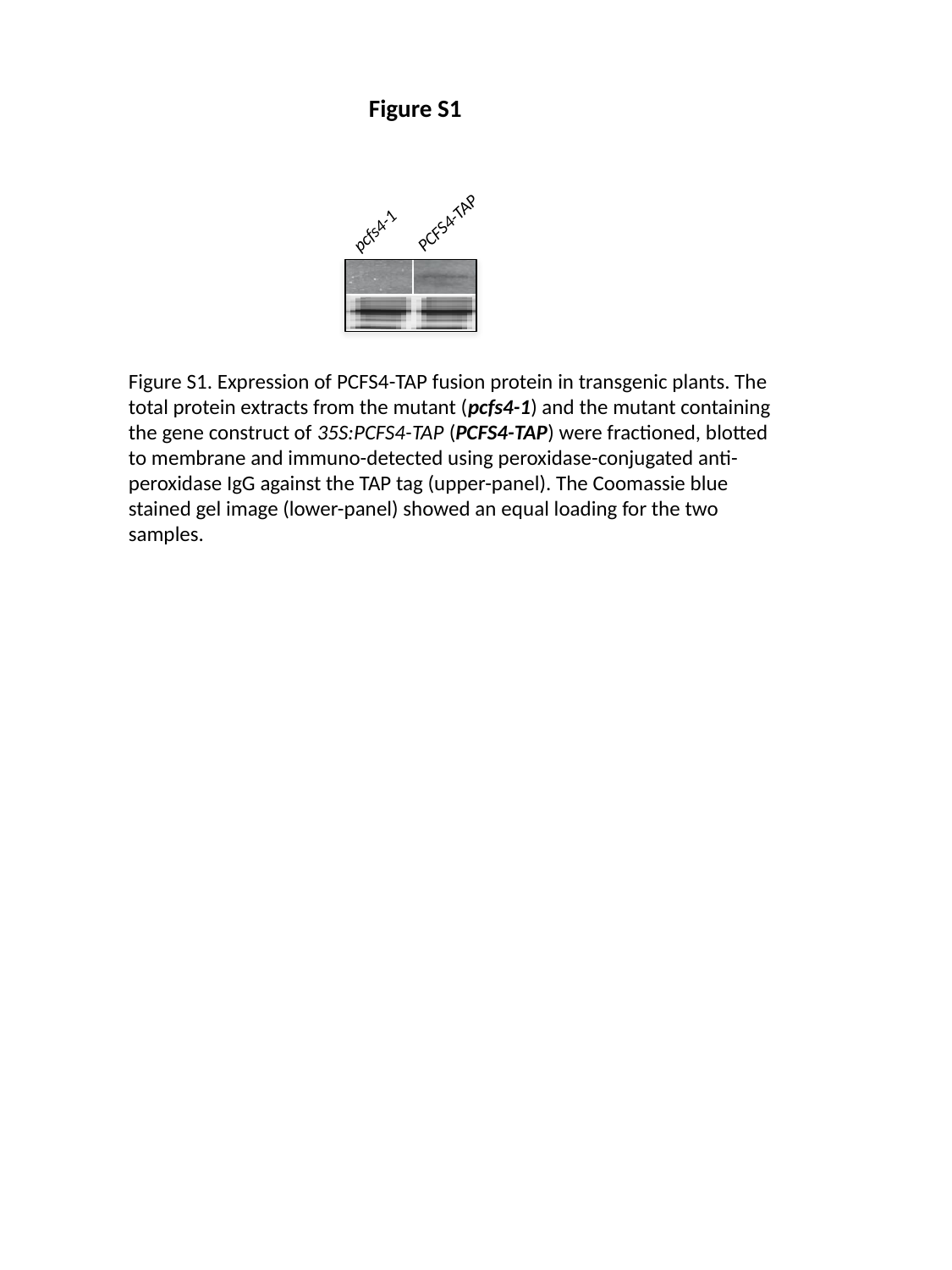

Figure S1
PCFS4-TAP
pcfs4-1
Figure S1. Expression of PCFS4-TAP fusion protein in transgenic plants. The total protein extracts from the mutant (pcfs4-1) and the mutant containing the gene construct of 35S:PCFS4-TAP (PCFS4-TAP) were fractioned, blotted to membrane and immuno-detected using peroxidase-conjugated anti-peroxidase IgG against the TAP tag (upper-panel). The Coomassie blue stained gel image (lower-panel) showed an equal loading for the two samples.

## Slide 2
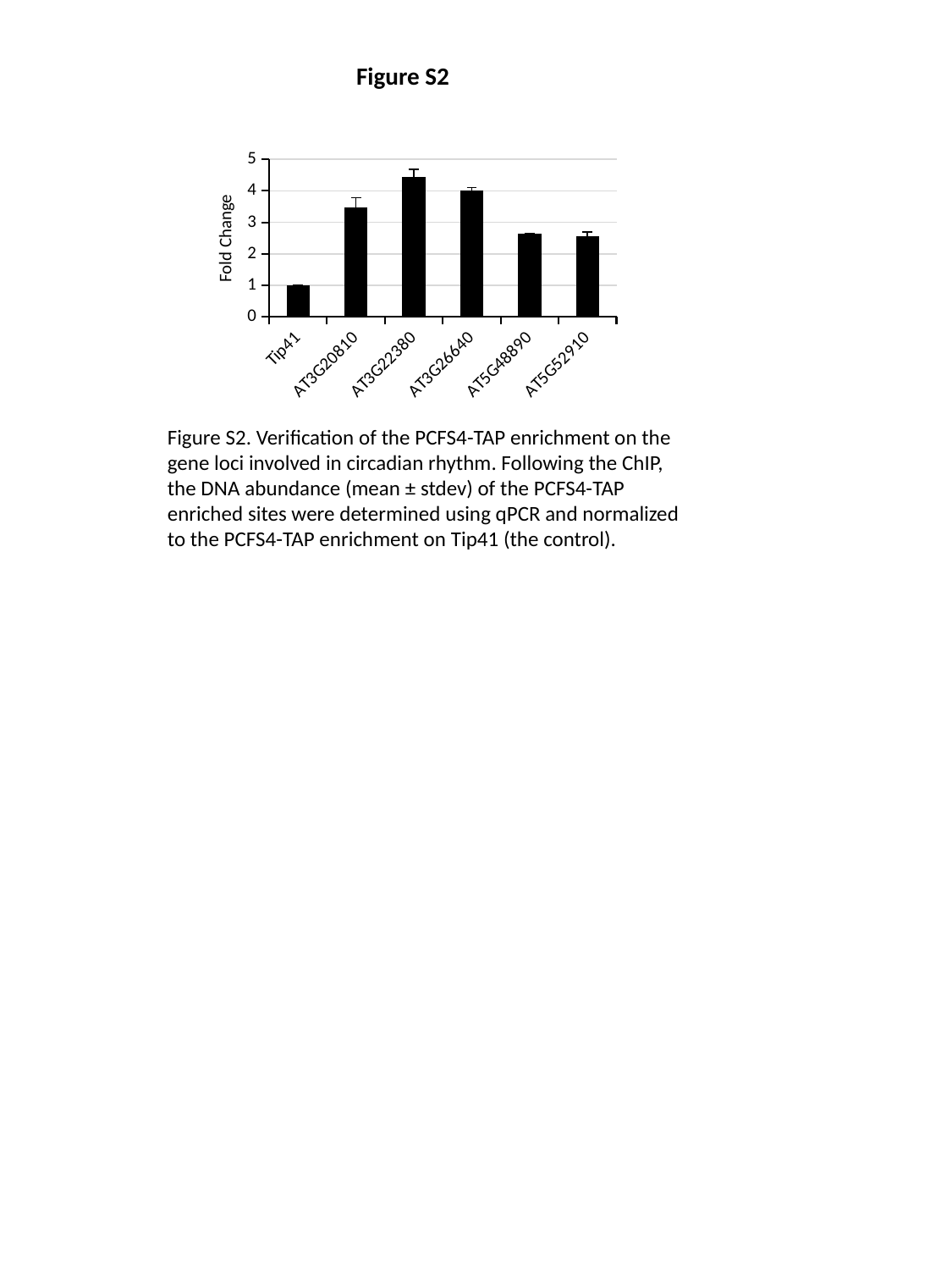

Figure S2
### Chart
| Category | |
|---|---|
| Tip41 | 1.0 |
| AT3G20810 | 3.482202253184498 |
| AT3G22380 | 4.43827788827138 |
| AT3G26640 | 3.999999999999995 |
| AT5G48890 | 2.639015821545787 |
| AT5G52910 | 2.549121254638526 |Fold Change
Figure S2. Verification of the PCFS4-TAP enrichment on the gene loci involved in circadian rhythm. Following the ChIP, the DNA abundance (mean ± stdev) of the PCFS4-TAP enriched sites were determined using qPCR and normalized to the PCFS4-TAP enrichment on Tip41 (the control).

## Slide 3
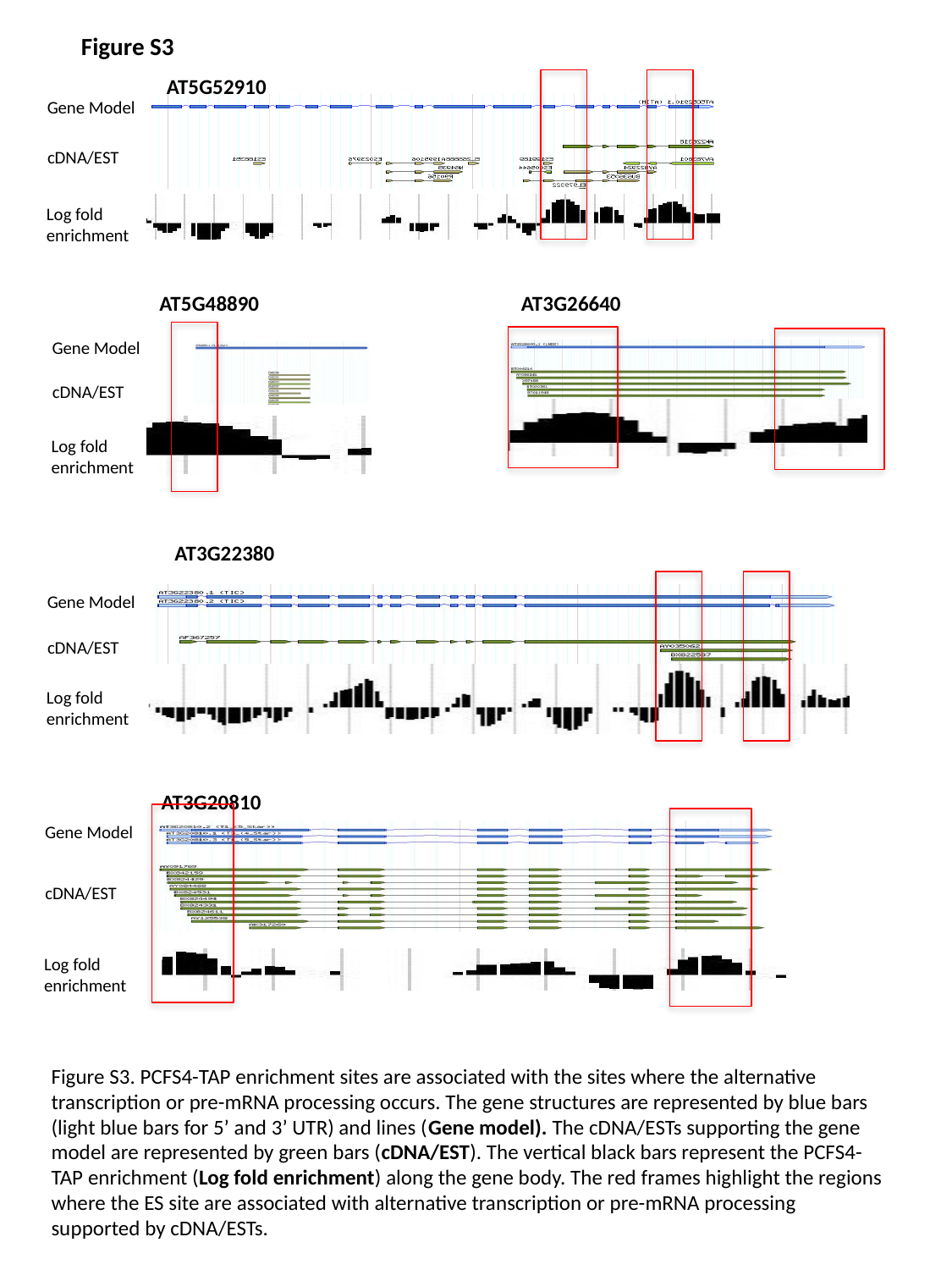

Figure S3
AT5G52910
Gene Model
cDNA/EST
Log fold enrichment
AT5G48890
AT3G26640
Gene Model
cDNA/EST
Log fold enrichment
AT3G22380
Gene Model
cDNA/EST
Log fold enrichment
AT3G20810
Gene Model
cDNA/EST
Log fold enrichment
Figure S3. PCFS4-TAP enrichment sites are associated with the sites where the alternative transcription or pre-mRNA processing occurs. The gene structures are represented by blue bars (light blue bars for 5’ and 3’ UTR) and lines (Gene model). The cDNA/ESTs supporting the gene model are represented by green bars (cDNA/EST). The vertical black bars represent the PCFS4-TAP enrichment (Log fold enrichment) along the gene body. The red frames highlight the regions where the ES site are associated with alternative transcription or pre-mRNA processing supported by cDNA/ESTs.
